# New Appliances and Things Medical

**Published:** 1903-12-26

**Authors:** 


### NEW APPLIANCES AND THINGS MEDICAL.

### BANANINE FLOUR AND BREAD.

(Joseph Appleby and Sons, Limited, Liverpool.)

Banana flour is made from dried unripe bananas, and forms a highly nutritious and assimilable preparation. The idea is current that this flour is deficient in proteid, but such is not the case for it contains nearly twice as much as ordinary wheat flour does, besides being rich in carbohydrates, fats, and mineral matter. It has the pleasant flavour and aroma of the banana, and can form the chief ingredient of many agreeable household recipes. We have examined a sample of Bananine bread made from this flour. The loaf proved most palatable and possesses the acceptable property of keeping fresh for many days. Considering the high nutritive value of these products, they should form a useful addition to our dietary.

